# Reducing computational cost of large-scale genomic evaluation by using indirect genomic prediction

**DOI:** 10.3168/jdsc.2021-0097

**Published:** 2021-08-20

**Authors:** S. Tsuruta, D.A.L. Lourenco, Y. Masuda, T.J. Lawlor, I. Misztal

**Affiliations:** 1 Animal and Dairy Science Department, University of Georgia, Athens 30602; 2 Holstein Association USA Inc., Brattleboro, VT 05301

## Abstract

Over half a million Holsteins are being genotyped annually in the United States. The computational cost of including all genotypes in single-step genomic (ssG)BLUP is high, although it is feasible to conduct large-scale genomic prediction using an efficient algorithm such as APY (algorithm for proven and young). An effective method to further reduce the computing cost could be the use of indirect genomic predictions (IGP) for genotyped animals when they have neither progeny nor phenotypes. These young genotyped animals have no effect on the other genotyped animals and could have their genomic prediction done indirectly. The main objective of this study was to calculate IGP for various groups of genotyped animals and investigate the reduction in computing time as well as bias and accuracy of the IGP. We compared IGP with genomic (G)EBV for 18 linear type traits in US Holsteins, including 2.3 million (M) genotyped animals. The full data set consisted of 10.9M records for 18 linear type traits up to 2018 calving, 13.6M animals in the pedigree, and 2.3M animals genotyped for 79K SNP. For IGP, ssGBLUP included all genotyped animals except those with neither progeny nor phenotypes by year from 2014 to 2018 (i.e., the target animals). The SNP marker effects were computed based on GEBV for genotyped animals that had progeny, or phenotypes, or both. Further, IGP were calculated for target genotyped animals in each year group. For all genotyped animal groups from 2014 to 2018, the coefficients of determination (R^2^) of a linear regression of GEBV on IGP were 0.960 for males and 0.954 for females for 18 traits on average. To reduce computing costs, the SNP marker effects were calculated based on GEBV from randomly selected genotyped animals from 15K to 60K. By randomly selecting a small number of genotyped animals, the computing time was dramatically reduced. As more genotyped animals were randomly selected to calculate SNP effects, R^2^ was higher (more accurate) and the regression coefficient was lower (more inflated IGP). In a practical genomic evaluation in US Holsteins, to get sufficient contributions from GEBV, 25K to 35K is a rational number of genotyped animals that can be randomly selected to compute SNP effects and obtain accurate and unbiased IGP. Considering the computing time and both unbiasedness and accuracy of IGP, genomic evaluation can be conducted separately in GEBV for genotyped animals with phenotypes or progeny and in IGP for young genotyped animals. This can be a practical solution when conducting a large-scale genomic evaluation and would enable more frequent evaluation at lower cost, especially when many genotyped animals have neither phenotypes nor progeny.

## Highlights

• Genomic evaluation is expensive with a large number of genotyped animals.
• Indirect genomic prediction dramatically reduces the computing cost by using randomly selected genotyped animals.
• Indirect genomic evaluations are accurate and unbiased.

## Graphical Abstract

**Figure:**
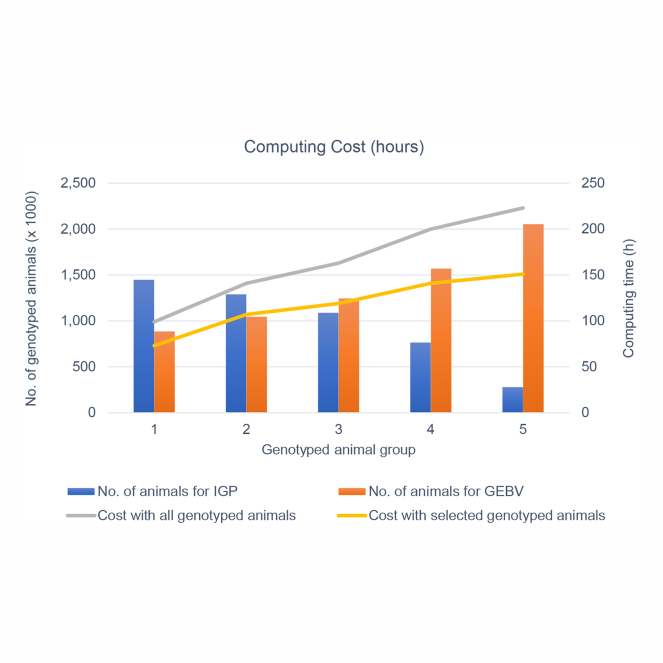
**Summary:** In the US dairy population, the number of genotyped animals has increased rapidly over the decade. The computation of large-scale genomic evaluations can be highly expensive, especially when conducting more frequent evaluations. One solution to reduce time and cost could be the use of indirect genomic prediction. We investigated how indirect prediction should be conducted and discovered a practical approach to calculate accurate and unbiased indirect genomic predictions using SNP marker effects from a small number of randomly selected genotyped animals. The results of this study can be applicable and useful in other breeds and species.

Heavy computation is inevitable in large-scale genomic evaluations. Since the national genomic evaluation for US Holsteins started in 2009, the number of genotyped animals has increased considerably. When conducting genomic prediction for a large number of genotyped animals, solving large mixed model equations (**MME**) is the most time-consuming process. Constructing the inverse of the combined relationship matrix (**H**^−1^)—which is composed of the inverse of the pedigree-based relationship matrix (**A**^−1^) for all animals, the inverse of the genomic relationship matrix (**G**^−1^), and the inverse of the pedigree-based relationship matrix $\left(A_{22}^{-1}\right)$ for all genotyped animals (Aguilar et al., 2010; Christensen and Lund, 2010)—is another key process in single-step genomic BLUP (**ssGBLUP**). The **A**^−1^ can be recursively obtained with Henderson's method (Henderson, 1976; Hudson et al., 1982), and the matrix by vector multiplication by iteration on data is highly efficient without constructing **A**^−1^ in solving the MME for a large number of animals. In addition, $A_{22}^{-1}$ can be calculated efficiently (Strandén and Mäntysaari, 2014; Masuda et al., 2017; Strandén et al., 2017). However, calculation of **G**^−1^ is expensive using a direct inversion method, even with sparse matrix operations (Pérez-Enciso et al., 1994; Masuda et al., 2015). With reference to Henderson's method, Misztal et al. (2014a) and Fragomeni et al. (2015) proposed a recursive method called the algorithm of proven and young animals (**APY**). This method calculates the approximated **G**^−1^ for a large number of noncore genotyped animals based on the direct **G**^−1^ for a minimum number of core genotyped animals, assuming that these core animals represent most of the independent chromosome segments in the genome. As of 2020, the number of genotyped US Holsteins has reached over 3 million, with half a million genotyped animals being added every year (CDCB, 2020). Even using the APY method, the computational cost is still high when including all genotyped animals. This could create a bottleneck in the future, especially when conducting frequent evaluations (e.g., every month or every week). One way to overcome issues with computing cost is to remove old genotyped animals that have been already culled and had neither progeny, nor phenotypes, nor semen stock (Koivula et al., 2018). However, when millions of animals are genotyped in several years, removing old genotyped animals will not be a substantial solution to the heavy computation required in genomic evaluation. Another option is to remove the young genotyped animals with neither progeny nor phenotypes from the ssGBLUP and calculate indirect genomic predictions (**IGP**) for those animals. If SNP effects based on genomic (**G**)EBV for other animals have sufficient genomic information, IGP for those young genotyped animals can be calculated by a linear function of SNP effects and contents. Therefore, it may be more practical to predict their genomic performance separately and indirectly, rather than to include them in ssGBLUP evaluations to obtain GEBV.

About 90% of the genotyped animals included in the genomic evaluation for type traits in US Holsteins are young females (heifers), which may have neither phenotypes for type traits nor progeny in the future, although they may have other phenotypes (e.g., production traits; Tsuruta et al., 2021). Including all of these animals in the main routine evaluation may not be reasonable because of the computing cost. Garcia et al. (2020) reported that the accuracy of IGP was as high as that of GEBV in American Angus data, where about 70% of the genotyped animals had phenotypes. In that study, they did not investigate which animals should be included in the computation of GEBV and IGP or how GEBV for those genotyped animals affect accuracy and bias in IGPs when those genotyped animals have neither phenotype nor progeny. When genotyped animals have phenotypes, those phenotypes should be used to calculate GEBV; therefore, those genotyped animals should not be the target for IGP. In addition, the total computing cost to obtain GEBV and IGP is unknown. The IGP, which is the genomic prediction for genotyped animals indirectly calculated from SNP marker effects based on GEBV for other genotyped animals, could be a practical choice to reduce the computational cost in genomic evaluations. This is true if the IGP has no impairment in accuracy and bias. The objective of this study was to investigate a practical approach to calculating IGP to reduce the computational cost without deteriorating accuracy and bias in genomic predictions for a large number (i.e., over 2 million) of genotyped animals in US Holsteins using 18 linear type traits.

Phenotypes for 18 linear type traits and pedigree information used in the 2018 genetic evaluation were provided by the Holstein Association USA Inc. (Brattleboro, VT). Genotypes up to 2018 were provided by the Council on Dairy Cattle Breeding (Bowie, MD). The full data set consisted of 10,946,264 repeated records for 7,216,767 cows, including 7,044,210 cows with no genotypes up to 2018 calving, 13,591,145 animals in the pedigree, and 2,334,951 animals genotyped for 79,294 SNP. Different start dates (ranging from 2014 until 2018) based on year of birth were used to create 5 different sets of genotyped animals. Genotyped animals with phenotypic records or progeny were included in each genomic data set to obtain GEBV with the ssGBLUP. Genotyped animals with neither progeny nor phenotypes and born after the start date would obtain IGP using the SNP effects based on the GEBV for other animals with phenotypes or progeny. First, genomic prediction was conducted via ssGBLUP using the full data set (i.e., benchmark). When calculating GEBV, 20K genotyped animals were randomly chosen as core animals for APY. The MME for the 18-trait animal model (Tsuruta et al., 2002) was solved via the BLUP90IOD program, which uses the preconditioned conjugate gradient method by iteration on data (Tsuruta et al., 2001). The convergence criterion of 10^−12^ based on relative adjusted right-hand sides (Tsuruta et al., 2001) was used. The program was originally created to calculate BLUP and revised with the ssGBLUP feature later. Second, ssGBLUP was conducted to predict GEBV using each genomic data set from 2014–2018 to 2018, in addition to all phenotypes and pedigree information as described before. Third, SNP effects were calculated via POSTGSF90 (Aguilar et al., 2014; Misztal et al., 2014b) from the GEBV for each genomic data set based on the formula (Wang et al., 2012) $\hat{u}=\lambda DZ^{\prime }G^{-1}\hat{a},$ where $\hat{u}$ is a vector of SNP marker effects, $\lambda =\frac{\sigma_{u}^{2}}{\sigma_{a}^{2}},$ where $\sigma_{u}^{2}$ and $\sigma_{a}^{2}$ are the variances of **u** and **a**, respectively; **D** = **I**; **Z** is a matrix of genotypes with the dimension of the target genotyped animals by the number of SNPs; and $\hat{a}$> is a vector of additive genetic effects (GEBV). When calculating SNP effects by GEBV using all genotyped animals with phenotypes or progeny in each year group, the same 20K core animals were used for APY. In contrast, when calculating SNP effects by GEBV using randomly selected genotyped animals from those animals, ranging from 15K to 60K, the APY was not needed (i.e., no core animals), so these animals were not the same animals as in the core previously used for APY to obtain GEBV. Last, IGP for other genotyped animals, which had neither progeny nor phenotypes (i.e., young bulls or heifers) in 2014–2018, 2015–2018, 2016–2018, 2017–2018, and 2018 were calculated from the SNP marker effects as $IGP=Z\hat{u}$ (Strandén and Garrick, 2009; Garcia et al., 2020) via PREDF90 (Misztal et al., 2014b). This calculation of IGP requires accurate SNP estimates from the GEBV. To calculate the SNP marker effects, all genotyped animals or a small number of randomly selected genotyped animals can be used (Lourenco et al., 2015). In a nutshell, the steps of this process for the 2014–2018 genomic data set, for example, involved the computation of GEBV using 38% of all genotyped animals (886,176) and all nongenotyped animals with phenotypes and progeny available in ssGBLUP (Table 1). Next, SNP marker effects were calculated based on GEBV from those genotyped animals or random subset thereof. Finally, IGP for the remaining 62% of the genotyped animals (1,448,775) that had neither progeny nor phenotypes were computed by those SNP effects (Table 1). Likewise, in the 2018 genomic data, 88% of the genotyped animals directly received GEBV, all or a randomly selected subset of them were used to estimate SNP effects, and 12% of the genotyped animals were used to estimate IGP.

**Table 1:** Numbers of genotyped animals used for the computation of genomic prediction (GEBV) and indirect genomic prediction (IGP), computing time in hours, and number of iterations (ITR) to converge for each genotyped data set

YOB^1^	No. of IGP			No. of GEBV	No. of IGP/no. of GEBV (%)	Computing time^2^ (h)			No. of ITR
	Male	Female	Total			GEBV	IGP	Total	
2014–2018	144,602	1,304,173	1,448,775	886,176	62	72	27 − 5 (1)	99 − 77 (73)	1,003
2015–2018	117,550	1,171,398	1,288,948	1,046,003	55	106	35 − 8 (1)	141 − 114 (107)	1,052
2016–2018	89,025	1,000,120	1,089,145	1,245,806	47	118	45 − 10 (1)	163 − 128 (119)	1,101
2017–2018	57,699	707,181	764,880	1,570,071	33	140	60 − 12 (1)	200 − 152 (141)	1,177
2018	23,217	257,044	280,261	2,054,690	12	153	70 − 13 (1)	223 − 166 (151)	1,332
All	—	—	—	2,334,951	—	—	—	177	1,433

1 YOB = years of birth for genotyped animals included in IGP defined as genomic data groups.

2 Computing times in parentheses are when randomly selected genotyped animals were used to calculate IGP. In computing times for IGP and Total, “− X” indicates hours without creating the inverse of genomic relationship matrix.

Table 1 shows computing time (wall-clock time) in hours using 4 CPU cores and the number of iterations for genomic data sets 2014–2018, 2015–2018, 2016–2018, 2017–2018, and 2018. Strictly speaking, computing time can vary depending on the computational environment, such as software, hardware, and sharing conditions. With the full data set, the computing time for GEBV was 177 h with 1,433 iterations. The computing time was mostly consumed by calculating **G**^−1^ with APY (26 h) and solving the MME (150 h). As the number of genotyped animals for GEBV increased, the computing time for IGP in Table 1 increased substantially due to the heavy calculation of SNP effects in $\hat{u}=\lambda DZ^{\prime }G^{-1}\hat{a},$ when computing **G**^−1^, even with APY for a large number of genotyped animals in **a**. On the other hand, the matrix-vector multiplication in IGP = **Zu** took a few minutes for all data sets. The total computing time for calculations of GEBV and IGP ranged from 99 h for 2014–2018 to 223 h for 2018. In this study, the POSTGSf90 program was used to calculate SNP marker effects, and about 80% of the computing time was spent to create **G**^−1^. However, if the SNP prediction is implemented inside the BLUP90IOD2 program, this additional computing time can be saved by avoiding creating the same **G**^−1^ twice. Another choice is to store **G**^−1^ as an output file when running BLUP90IOD2 and to read it again with POSTGSF90. Table 1 also shows the computing time for IGP when excluding the computing time of creating **G**^−1^. For example, in the 2018 data, it took 70 − 13 = 57 h to calculate **G**^−1^ with APY. By subtracting these hours from 223 h, now the computing time for IGP is 166 h, which is below the 177 h for the full data set.

To further reduce the computing time for the calculation of IGP, SNP marker effects were predicted from only a portion of the genotyped animals in **a**; that is, randomly selected from 38% of all genotyped animals (i.e., 886,176 from 2014 to 2018): 15K, 20K, 25K, 30K, 35K, 40K, 45K, 50K, 55K, and 60K. The computation of **G**^−1^ for those small number of animals in **a** was fast, even with the direct inversion, and the corresponding computing time of SNP effects took less than 1 h using any number of selected genotyped animals from 15K to 60K. As a result, the total computing time was reduced from 99 h to 73 h for the 2014–2018 data and from 223 h to 151 h for the 2018 data (Table 1).

Table 2 shows b_0_ and b_1_ on the regression model fitting GEBV = b_0_ + b_1_ × IGP for genotyped animals that had neither progeny nor phenotypes in each year group, and mean absolute differences (**MEAN**) and maximum absolute differences (**MAX**) between GEBV and IGP for the genomic data from 2014 to 2018 using SNP effects in IGP from randomly selected 30K genotyped animals. These values from the 2015–2018, 2016–2018, 2017–2018, and 2018 year groups are not shown in the table because they were similar to those from 2014–2018 (e.g., b_0_ ranging from 1.2 to 1.4 for males and from 1.2 to 1.3 for females). The MEAN values were similar to b_0_ values except for traits with small or negative genetic gains. The sum of the b_0_ value and b_1_ × IGP must be reported together to avoid bias with the interpretation of IGP. The correlations of MEAN, MAX, and b_0_ with standardized genetic progress (**ΔG**) from Tsuruta et al. (2021) were high (0.96 to 0.98) for all genomic data sets. To compare b_0_ and ΔG on the same scale, MEAN, MAX, and b_0_ were also standardized by dividing by each genetic standard deviation. The high positive correlations indicate underprediction of IGP when ΔG is greater or selection is more intense. This bias in IGP is attributable to the different genetic base for each trait, and it can be adjusted by the mean difference between GEBV and IGP (Lourenco et al., 2018). The whole calculation of GEBV is required to obtain the exact genetic difference; however, because the target genotyped animals for IGP from the 2014–2018 genomic data spanned 5 years, the adjustment based on b_0_ or the genetic gains will be simple, rational, and practical. Without adjusting the genetic base correctly, these genotyped animals with IGP cannot be ranked together with other animals with GEBV. In this case, these animals with IGP should be ranked separately. Table 2 also shows b_1_ values on the regression model, which is the scaling factor or the slope on IGP, using the genomic data from 2014 to 2018. The b_1_ values from the 2015 to 2018 genomic data groups were similar to those from 2014 to 2018 on average (not shown in Table 2), ranging from 0.99 to 1.01 for males and 0.99 to 1.00 for females. The scaling factor indicates inflation (deflation) of IGP when b_1_ <1.0 (>1.0). Overall, the average scaling factor showed no inflation or deflation, ranging from 0.99 to 1.01 on average for all data sets. However, b_1_ ranged from 0.94 to 1.06 for males and from 0.95 to 1.05 for females for the individual 18 traits. The correlation between b_1_ and ΔG was high (0.84), implying that IGP is more deflated or GEBV is more inflated when the ΔG is larger (i.e., more directional selection), and less deflated IGP or less inflated GEBV when the trait has an intermediate optimum or assortative mating is being practiced (Tsuruta et al., 2021). Table 2 also shows the coefficient of determination (R^2^) of the regression model and the correlation between R^2^ and ΔG for the genomic data from 2014 to 2018. The R^2^ were high (0.950 and 0.949 for males and females on average, respectively, equivalent to correlations from 0.975 and 0.974) and ranged from 0.908 to 0.975 for males and from 0.905 to 0.974 for females for 18 traits. The R^2^ did not change over the years by increasing the number of genotyped animals for calculation of GEBV, ranging from 0.95 to 0.97 for males and from 0.95 to 0.96 for females (R^2^ from 2015 to 2018 are not shown in Table 2). This indicates that IGP were accurate regardless of the number of genotyped animals used to compute GEBV (for genotyped animals with phenotypes or progeny) and IGP (for genotyped animals with neither phenotypes nor progeny). Negative correlations (−0.44 and −0.55 for males and females, respectively) between R^2^ and ΔG indicate that traits with more directional selection tend to have lower accuracy in IGP.

**Table 2:** Intercept (b_0_), regression coefficient b_1_, and R^2^ in genomic (G)EBV = b_0_ + b_1_ × IGP,^1^ mean absolute differences (MEAN) and maximum absolute differences (MAX) between GEBV and IGP, and correlations of ΔG^2^ with each parameter, using SNP estimates for randomly selected 30K genotyped animals from 2014 to 2018 genomic data for 18 type traits^3^

Trait	ΔG	b_0_		b_1_		R^2^		MEAN		MAX	
		Male	Female	Male	Female	Male	Female	Male	Female	Male	Female
Stature	1.55	1.70	1.60	1.01	1.00	0.962	0.953	1.71	1.60	2.39	2.42
Strength	0.71	0.85	0.80	0.97	0.97	0.965	0.960	0.85	0.80	1.46	1.48
Body depth	0.88	1.14	1.05	0.98	0.98	0.958	0.953	1.13	1.05	1.75	1.76
Dairy form	1.18	1.81	1.66	1.00	0.99	0.937	0.928	1.81	1.66	2.51	2.48
Rump angle	−0.02	−0.05	−0.03	0.95	0.95	0.971	0.969	0.11	0.11	0.62	0.73
Rump width	1.11	1.25	1.17	0.98	0.98	0.964	0.958	1.24	1.17	1.86	1.84
Rear legs side view	−0.02	0.02	0.01	0.95	0.96	0.977	0.974	0.09	0.09	0.57	0.61
Foot angle	1.17	1.34	1.25	1.00	1.01	0.953	0.945	1.34	1.25	1.85	1.88
Fore attachment	2.10	2.22	2.11	1.03	1.03	0.959	0.947	2.24	2.12	2.83	2.87
Rear udder height	2.19	2.38	2.27	1.05	1.05	0.959	0.943	2.42	2.29	3.01	3.03
Rear udder width	2.00	2.41	2.28	1.06	1.05	0.951	0.931	2.45	2.30	3.04	3.10
Udder cleft	1.31	1.59	1.52	0.99	0.98	0.955	0.949	1.59	1.51	2.13	2.17
Udder depth	1.51	1.35	1.29	0.98	0.99	0.966	0.960	1.34	1.29	1.90	2.01
Front teat placement	1.20	1.43	1.35	0.98	0.98	0.951	0.946	1.39	1.33	1.96	2.08
Teat length	−0.21	−0.18	−0.18	0.94	0.95	0.973	0.969	0.18	0.19	0.91	0.93
Rear legs rear view	1.00	1.24	1.15	1.00	1.00	0.946	0.932	1.24	1.15	1.75	1.78
Feet and legs	1.25	1.45	1.35	1.05	1.04	0.926	0.905	1.47	1.36	1.95	2.01
Rear teat placement	1.07	1.29	1.22	0.97	0.97	0.960	0.956	1.28	1.21	1.81	1.86
Mean	1.11	1.29	1.21	0.99	0.99	0.957	0.949	1.33	1.25	1.91	1.95
SD	0.68	0.75	0.72	0.03	0.03	0.012	0.017	0.70	0.66	0.72	0.71
Correlation (ΔG)^2^	—	0.98	0.98	0.84	0.84	−0.44	−0.55	0.97	0.98	0.96	0.96

1 Indirect genomic predictions.

2 Genetic progress (adapted from Table 3 in Tsuruta et al., 2021) as correlations with b_0_, b_1_, or R^2^.

3 The values of b_0_, ΔG, MEAN, and MAX were standardized by dividing by the genetic standard deviation for each trait.

As described before, 10 randomly selected genotyped animal groups were used to reduce the computing time for calculation of IGP in the 2014–2018 data (i.e., 15K, 20K, 25K, 30K, 35K, 40K, 45K, 50K, 55K, and 60K genotyped animals from 886,176). Figure 1 shows the average b_1_ for all 18 traits changing over these 10 animal groups. The b_1_ indicates slight inflation of IGP when all 886,176 genotyped animals in 2014–2018 were used. However, when randomly selected genotyped animals were used, IGP showed slight deflation to inflation when the number of animals increased from 15K to 60K, indicating that the genetic variance was larger in IGP than in GEBV as more genotyped animals were selected. When 25K genotyped animals were used, b_1_ was close to 1.0. This result suggests that a number of genotyped animals between 25K and 35K could be the appropriate range for IGP when we select genotyped animals randomly to calculate SNP marker effects. Figure 1 also shows the average R^2^ corresponding to b_1_ described above. When 35K or 40K genotyped animals were used to calculate IGP, the R^2^ reached the same level as the R^2^ from all 886,176 genotyped animals (i.e., 0.960 for males and 0.954 for females).

**Figure 1:**
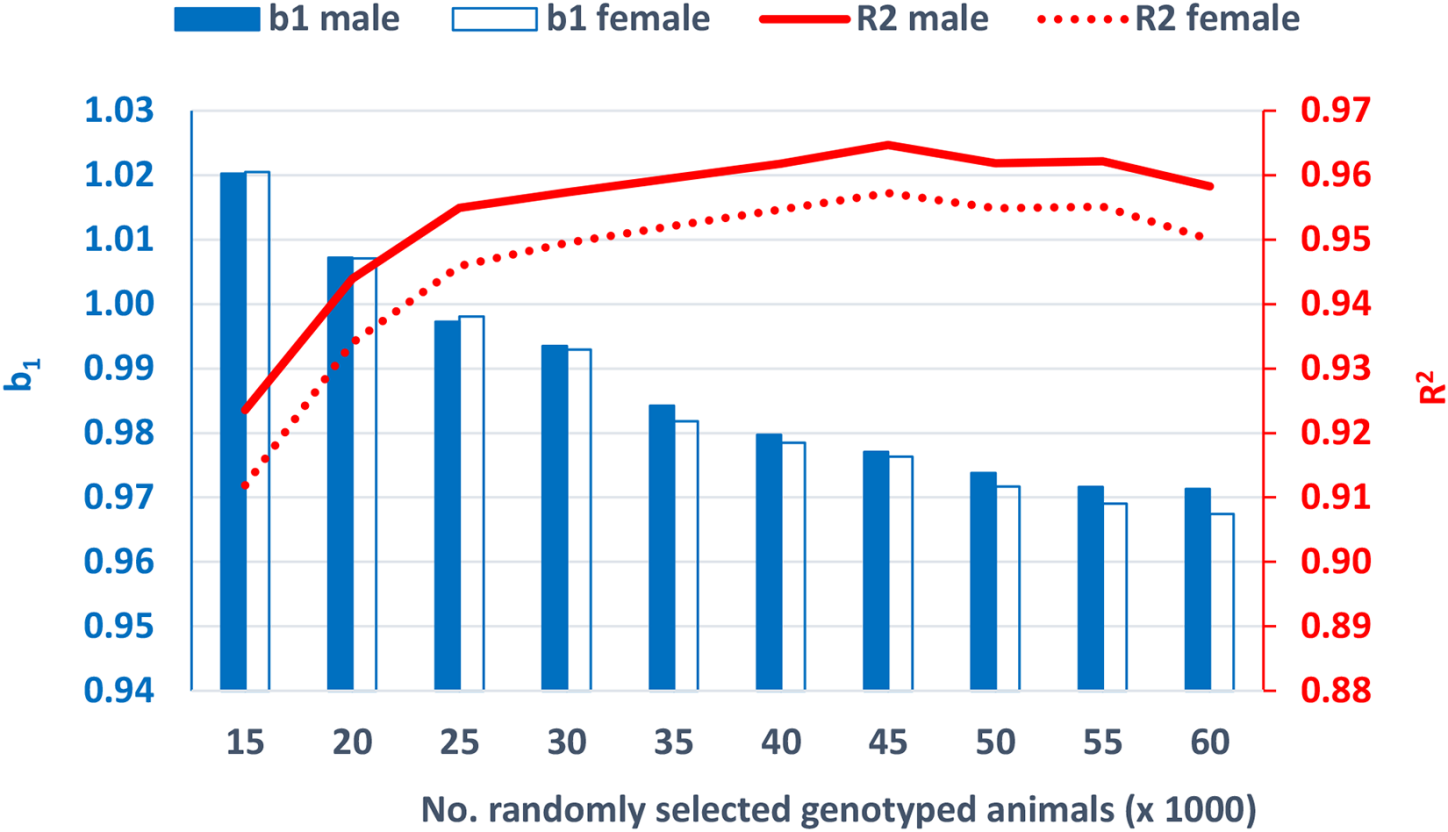
Changes in regression coefficient b_1_ and R^2^ in genomic (G)EBV = b_0_ + b_1_ × IGP (indirect genomic prediction) for randomly selected genotyped animals when computing SNP effects for 2014–2018 genomic data (b_0_ = intercept; b_1_ = blue bar for male and white bar for female; R^2^ = solid red line for male and dotted red line for female).

These numbers from 25K to 35K could be considered appropriate as the number of core animals in APY. In our study, the number of eigenvalues explaining 98% or 99% in the variation in **G** was around 20K, which means that 20K genotyped animals could provide sufficient information to accurately estimate the effects of all independent chromosome segments in this population. Therefore, 20K animals can be used as the core in APY under ssGBLUP evaluations. However, some of the selected core animals may provide redundant information (i.e., some of the animals may be highly correlated), possibly increasing the required number of core animals that represent all independent chromosome segments to a range from 25K to 35K. An additional comparison using the same 20K core animals from APY to obtain GEBV and calculate SNP marker effects was conducted. However, no difference was found using these 20K core animals and the randomly selected 20K animals; therefore, the results are not presented.

Considering the computing time and both biasedness and accuracy in IGP, genomic evaluations for a large number of genotyped animals can be conducted separately in GEBV and IGP. A small number of randomly selected genotyped animals can be used to accurately estimate SNP marker effects and can dramatically reduce the computational cost for IGP. The results of this study describe a practical solution when conducting a large-scale genomic evaluation and can make more frequent evaluations less costly.
